# Increased CCR4+ and Decreased Central Memory CD4+ T Lymphocytes in the Background Gastric Mucosa of Patients Developing Gastric Cancer After Helicobacter pylori Eradication: An Exploratory Study

**DOI:** 10.7759/cureus.31713

**Published:** 2022-11-20

**Authors:** Masaya Iwamuro, Takahide Takahashi, Araki Hirabata, Takehiro Tanaka, Fumio Otsuka, Horoyuki Okada

**Affiliations:** 1 Department of Gastroenterology and Hepatology, Okayama University Hospital, Okayama, JPN; 2 Division of Medical Support, Okayama University Hospital, Okayama, JPN; 3 Department of Pathology, Okayama University Hospital, Okayama, JPN; 4 Department of General Medicine, Okayama University Graduate School of Medicine, Dentistry, and Pharmaceutical Sciences, Okayama, JPN; 5 Department of Gastroenterology and Hepatology, Okayama University Graduate School of Medicine, Dentistry, and Pharmaceutical Sciences, Okayama, JPN

**Keywords:** carcinogenesis, lymphocytes, helicobacter pylori, gastric adenocarcinoma, flow cytometry

## Abstract

The composition of lymphocytes in the gastric mucosa following the eradication of *Helicobacter pylori* (*H. pylori*) in patients with and without gastric cancer has not been compared. This study performed a single spot analysis of gastric mucosal lymphocytes after *H. pylori *eradication in patients with (n = 13) and without (n = 20) gastric cancer. Our comprehensive analysis of lymphocyte composition in the gastric mucosa revealed that: i) the proportion of CD8^+^/CD3^+^ cells was relatively higher in the peri-tumor mucosa than in the background mucosa; ii) the proportion of CCR4^+^/CD3^+^ cells was higher, and the ratio of CD62L^+^/CD3^+^CD4^+^ cells was relatively lower in the gastric mucosa of cancer patients than in non-cancer patients; and iii) the proportion of CD45RA^−^CD62L^+^/CD3^+^CD4^+^ cells, namely, the central memory CD4^+^ T-cell fraction, was lower in the gastric mucosa of cancer patients than in non-cancer patients. Although the exact mechanism of the altered proportions of CCR4^+^/CD3^+^ and central memory CD4^+^ cells in the gastric mucosa of patients with cancer is unknown, focusing on lymphocytes in the gastric mucosa might help improve our understanding of gastric cancer development after *H. pylori* eradication.

## Introduction

It is well known that *Helicobacter pylori* infection causes inflammatory cell infiltration in the stomach. Chronic inflammation in the stomach is considered to lead to sequential histological alterations in the gastric epithelium, such as atrophic gastritis, intestinal metaplasia, dysplasia, and cancer [1,2]. Although the eradication of *H. pylori* reduces the risk of carcinogenesis, gastric cancer (GC) development is still observed in some patients [3-5]. It is now recognized that immune cells play a significant role in the growth, invasion, and initiation of GC. T lymphocytes are the most enriched immune cell population in the tumor microenvironment of the stomach [1,6-8].

Our earlier study compared the histological findings of patients who developed early GC after successful *H. pylori* eradication and those who did not develop a gastric neoplasm over three years after successful *H. pylori* eradication [9]. After propensity score-matching for sex, age, and years after successful eradication, multivariate analysis revealed that inflammation of the greater curvature of the antrum (risk ratio: 5.92, 95% confidence interval: 2.11-16.6) and the lesser curvature of the corpus of the stomach (risk ratio: 3.56, 95% confidence interval: 1.05-13.2) were independent risk factors [9]. Based on these results, we hypothesized that persistent chronic inflammation of the gastric mucosa, even after *H. pylori* eradication, is involved in the development of GC.

Based on findings from prior studies showing that T lymphocytes play a specific role in initiating and promoting the transformation of epithelial cells into precancerous cells, we hypothesized that the lymphocyte composition of the stomach may be key to identifying patients susceptible to GC development. Such technology will enable efficient mass screening and early detection of GC after *H. pylori* eradication. Therefore, we performed a single spot analysis of the gastric mucosal T lymphocytes after *H. pylori* eradication in patients with and without early GC using the lymphocyte isolation technique from an endoscopic biopsy fragment [10-12]. This study aimed to determine the differences between the composition of T-lymphocytes in the stomach of patients with and without GC and the differences between T lymphocyte populations of the peri-tumor and background gastric mucosa.

## Materials and methods

Patients

Flow cytometry was prospectively performed between March 2020 and April 2021 at Okayama University Hospital (Okayama, Japan) on endoscopic biopsy specimens obtained from 14 patients with GC that developed after *H. pylori* eradication and 20 patients without GC. The inclusion criteria for patients with and without GC were as follows: (i) age 20 to 89 years; (ii) a history of *H. pylori* eradication, confirmed with the urea breath test; (iii) no prior history of surgical resection of the stomach; (iv) no history of GC treatment; (v) not taking immunosuppressive or anticancer drugs; (vi) no known autoimmune gastritis or inflammatory bowel disease. The exclusion criteria were as follows: (i) pregnancy, breastfeeding, or positive pregnancy test; (ii) known irreversible bleeding disorder; (iii) platelet count 50,000 cells/mm^3^ or known platelet dysfunction.

**Figure 1:**
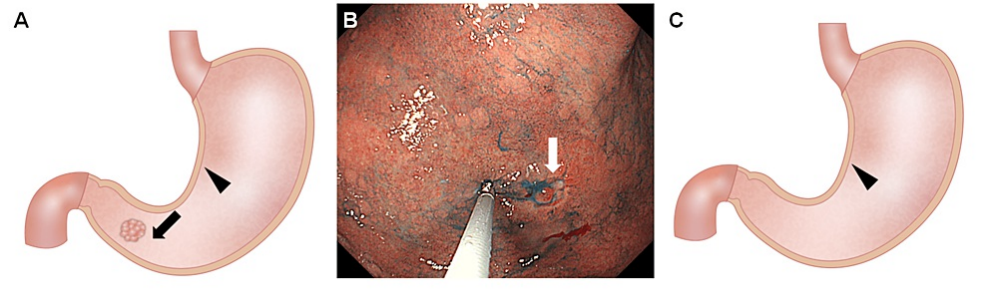
Schematic illustrations and an endoscopic image of biopsy sampling. A) A single specimen was obtained from the peri-tumor mucosa that was approximately 5 mm away from the cancer lesion (GC-peri-tumor sample, arrow, n = 14). Another specimen was obtained from the lesser curvature of the gastric body of the same patient (GC-body-sample, arrowhead, n = 14). B) Representative endoscopic image of the lesion in the GC patient. The GC-peri-tumor sample was obtained using biopsy forceps from the mucosa, approximately 5 mm away from the cancer lesion (arrow). C) A single specimen was obtained from the lesser curvature of the gastric body in patients without GC (non-GC-body sample, n = 20). GC: gastric cancer

In 14 GC patients, a single specimen was obtained from the peri-tumor mucosa, which was approximately 5 mm away from the GC lesion (GC-peri-tumor sample, Figures 1A-1B, arrow). Another specimen was obtained from the lesser curvature of the gastric body of the same patient (GC-body sample, Figure 1A, arrowhead). Although early GC was suspected at the referring hospital, the preoperative diagnosis was changed to advanced-stage cancer in one patient. Surgical resection revealed serosal invasion with multiple lymph node metastases (ypT4a, ypN3a, and stage IIIB). Therefore, this patient was excluded from this study. Pathological evaluation after resection revealed that GC was confined to the mucosal or submucosal layer in the remaining 13 patients.

In 20 patients without GC, a single specimen was obtained from the lesser curvature of the gastric body (non-GC-body sample, Figure 1C). Endoscopy and biopsy were performed as part of the standard care for neoplasia screening, mostly on a yearly basis.

One-step lymphocyte isolation and flow cytometric analysis

**Figure 2:**
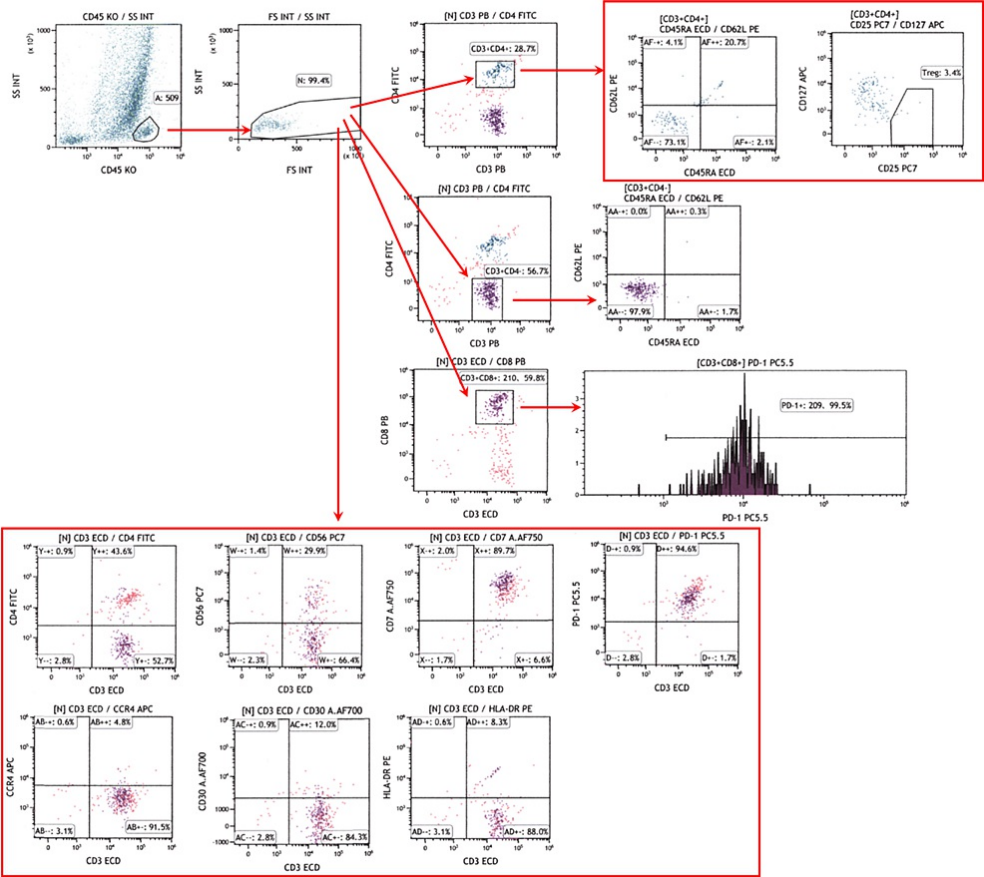
The gating strategy used in the present study An example of the GC-peri-tumor sample is shown.

Lymphocytes were isolated from a single biopsied specimen using a one-step lymphocyte isolation procedure, a modified version of our previously reported protocol [10-12]. Monoclonal antibodies against CD45 (clone J33, Beckman Coulter, Pasadena, CA, USA), CD4 (13B8.2; Beckman Coulter), CD3 (UCHT1; Beckman Coulter), CD25 (B1.49.9; Beckman Coulter), CD127 (R34.34; Beckman Coulter), CD45RA (2H4; Beckman Coulter), CD62L (DREG56; Beckman Coulter), CD8 (B9.11; Beckman Coulter), CD56 (N901; Beckman Coulter), CD7 (8H8.1; Beckman Coulter), PD-1 ( CD279; PD1.3; Beckman Coulter), CD30 (HRS4; Beckman Coulter), HLA-DR (Immu-357; Beckman Coulter), and CCR4 (CD194; L291H4; BioLegend) were used [13,14]. Immunostained cells were analyzed using FACScan (Navios flow cytometer, Beckman Coulter) and Kaluza analysis software, version 1.3 (Beckman Coulter). Lymphocytes were separated by flow cytometry based on high CD45 antigen expression and the forward- and side-scatter properties. Subsequently, flow cytometry data were analyzed according to the percentage of cell populations detected in each quadrant on two-dimensional scatterplots. We calculated the percentages of CD4^+^, CD8^+^, CD56^+^, CD7^+^, PD1^+^, CCR4^+^, CD30^+^, and HLADR^+^ cells among the CD3^+^ cells. We also assessed the percentages of regulatory T (Treg) cells, CD45RA^+^, and CD62L^+^ cells among CD3^+^CD4^+^ cells, the percentages of CD45RA^+^ and CD62L^+^ cells among CD3^+^CD4^−^ cells, and PD-1^+^ cells among CD3^+^CD8^+^ cells. In this study, we defined CD25^+^CD127^low/−^/CD3^+^CD4^+^ cells as the Treg fraction. An example of the gating strategy is presented in Figure 2.

Analysis

First, to reveal the differences between the lymphocyte population of the peri-tumor mucosa and that of the background gastric mucosa, we compared the flow cytometric results for GC peri-tumor (n = 14) and GC-body samples (n = 14). Second, to analyze the differences between the composition of lymphocytes in the stomach of patients with GC and those without GC, we compared the flow cytometric results of GC-body (n = 14) and non-GC-body samples (n = 20).

Statistical analyses

Statistical analyses were performed using the JMP 14.0.0 software (SAS Institute Inc., Cary, NC, USA). The F-test or t-test was used to compare patient characteristics. The Wilcoxon signed-rank test was used to compare GC-peri-tumor and GC-body samples, while the Wilcoxon rank-sum test was used to compare GC-body and non-GC-body samples. As this was an exploratory study, the sample size was not estimated. Statistical significance was set at p < 0.05.

Ethics approval

Patients were prospectively registered and analyzed in this study. Written informed consent was obtained from all participants. This study adhered to the principles of the Declaration of Helsinki and was approved by the ethics committee of the Okayama University Hospital. The study protocol was registered with the UMIN Clinical Trials Registry (UMIN000039863).

## Results

Patient characteristics

Characteristics of the enrolled patients are summarized in Table 1. Endoscopic biopsy specimens were obtained from 13 patients (11 men and two women) with GC that developed after *H. pylori* eradication and from 20 patients (10 men and 10 women) without GC. Although the difference was not statistically significant (p = 0.067), there was a male predominance in the GC group. The mean age was 66.0 years (range: 53-86 years) in GC patients and 64.3 years (range: 41-81) in the non-GC group, respectively. The mean period between *H. pylori* eradication and flow cytometric analysis was 5.5 years in the GC group and 7.2 years in the non-GC group. There were no significant differences between the two groups in terms of age, the period between *H. pylori* eradication and flow cytometric analysis, use of proton pump inhibitors, grade of gastric atrophy (closed vs. open type), or presence or absence of mucosal atrophy in the lesser curvature of the body.

**Table 1:** Clinical characteristics of the study patients. GC: gastric cancer; HP: *Helicobacter pylori*; SD: standard deviation.

	GC	non-GC	P value
Sex			
Male	11	10	0.067
Female	2	10	
Age (years, mean ± SD)	66.0 ± 8.5	64.3 ± 10.9	0.173
Years after HP eradication (mean ± SD)	5.5 ± 1.8	7.2 ± 4.5	0.141
Use of proton pump inhibitor			1.000
User	1	3	
Non-user	12	17	
Gastric atrophy			0.278
Closed type	3	9	
Open type	10	11	
Atrophy of the lesser curvature of the body			1.000
Present	12	18	
Absent	1	2	
Atrophy around the cancer lesion			
Present	13	NA	
Absent	0	NA	
Histological classification of gastric adenocarcinoma			
tub1	8	NA	
tub2	3	NA	
por2	1	NA	
Sig	1	NA	
Tumor location			
Body	6	NA	
Angle	2	NA	
Antrum	5	NA	
Tumor depth			
pT1a	12	NA	
pT1b1	1	NA	
Treatment for gastric cancer			
Endoscopic submucosal dissection	11	NA	
Surgery	2	NA	

GC was histologically classified as well-differentiated tubular adenocarcinoma (tub1, n = 8), moderately differentiated adenocarcinoma (tub2, n = 3), non-solid type poorly differentiated adenocarcinoma (por2, n = 1), and signet-ring cell carcinoma (sig, n = 1) and identified in the body (n = 6), angle (n = 2), and antrum (n = 5) of the stomach. The cancer was confined to the mucosa (T1a) of 12 patients, while the remaining patients had cancer with submucosal invasion within 0.5 mm from the muscularis mucosae (T1b1). Regarding the treatment, 11 patients underwent endoscopic submucosal dissection, and two patients (one patient with por2 and the other patient with sig GC) underwent surgical resection.

Comparison between the GC-peri-tumor and GC-body samples

**Figure 3:**
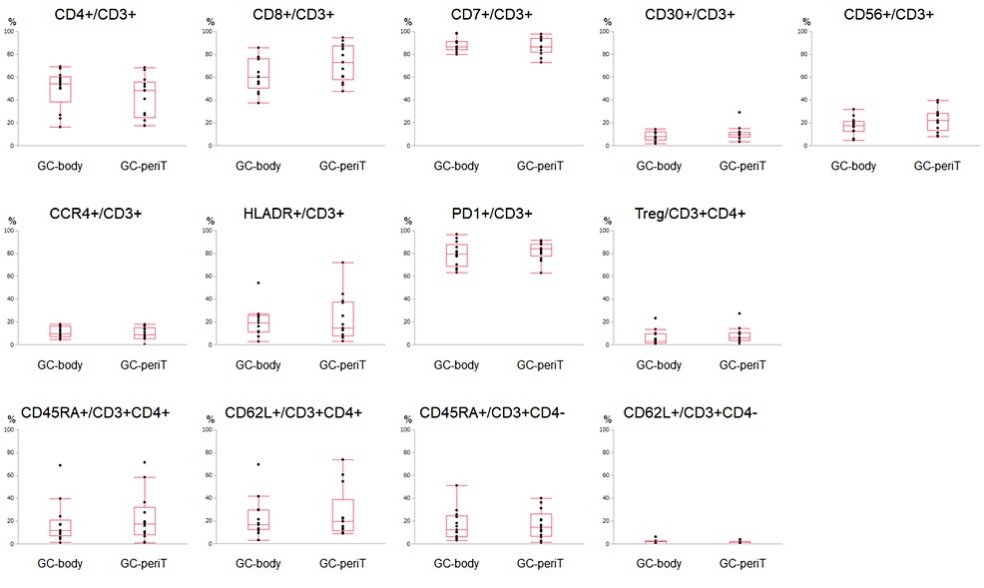
Flow cytometry results comparing the GC-peri-tumor (GC-periT) and GC-body samples. The GC-peri-tumor sample was obtained from the peri-tumor mucosa, which was approximately 5 mm away from the GC lesion. The GC-body sample was obtained from the lesser curvature of the gastric body of the same patient. No significant differences were observed between samples (Wilcoxon signed-rank test). Regulatory T (Treg) cells were defined as CD3^+^CD4^+^CD25^+^CD127^low/-^ cells. GC: gastric cancer

Results of the flow cytometric analysis are shown in Figure 3. There were no significant differences between the lymphocyte compositions of the GC peri-tumor and GC-body samples. However, the percentage of CD8^+^/CD3^+^ cells was relatively higher in the GC-peri-tumor (72.6 ± 16.2%) than in the GC-body samples (61.2 ± 14.4%) (p = 0.057).

According to the Laurén classification, 11 patients had intestinal-type GC, and the other two patients had diffuse-type GC [15]. Subanalysis comparing the GC-peri-tumor and GC-body samples in intestinal-type cancers (n = 11) revealed a significant difference between the percentage of CD8^+^/CD3^+^ cells in the GC-peri-tumor samples (76.4 ± 14.3%) and that of the GC-body samples (61.2 ± 14.4%) (Figure 7 in Appendices).

Comparison between the GC-body and non-GC-body samples

**Figure 4:**
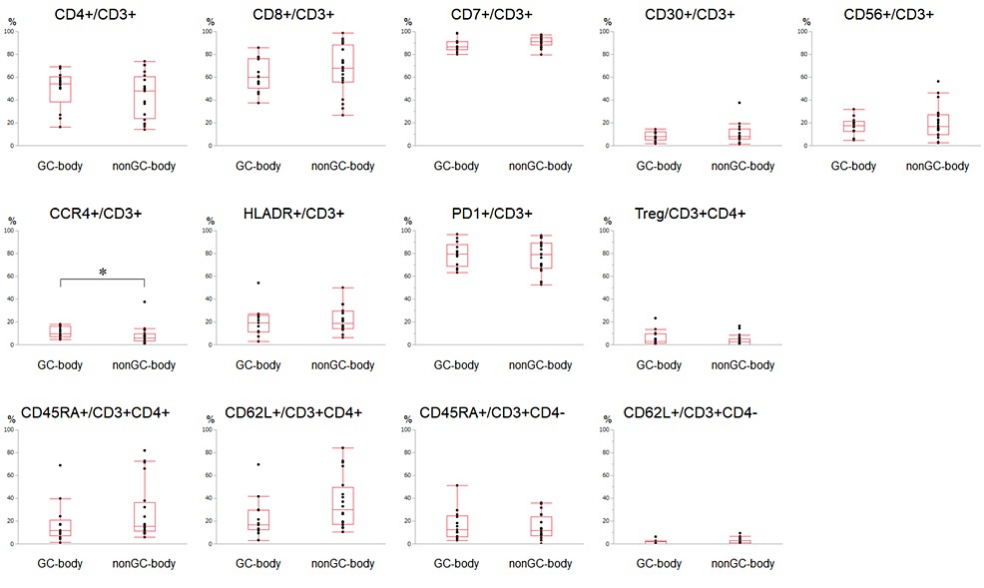
Flow cytometry results comparing the GC-body and non-GC-body samples. The GC-body sample was obtained from the lesser curvature of the gastric body of the patient with early GC. The non-GC-body sample was obtained from the lesser curvature of the gastric body of the patients without GC. Regulatory T (Treg) cells were defined as CD3^+^CD4^+^CD25^+^CD127^low/-^ cells. GC: gastric cancer *p < 0.05 (Wilcoxon rank-sum test).

The lymphocyte composition in the gastric body of GC patients (GC-body) and non-GC patients (non-GC-body) is shown in Figure 4. A comparison between the two groups revealed that the ratio of CCR4^+^/CD3^+^ cells was higher in the GC-body (11.1 ± 4.7%) than in the non-GC-body (7.7 ± 1.8%). In addition, although the difference was not statistically significant, the ratio of CD62L^+^/CD3^+^CD4^+^ cells was lower in the GC-body (22.8 ± 17.2%) than in the non-GC-body samples (36.3 ± 22.7%) (p = 0.065). No significant differences were observed for the other surface markers.

Subanalysis between the GC-body samples of the intestinal type (n = 11) and non-GC-body samples again showed that the percentage of CCR4^+^/CD3^+^ cells was higher in the GC-body (11.4 ± 5.0%) than in the non-GC-body (7.7 ± 8.1%) (Figure 8 in Appendices).

Central memory T cells among lymphocytes in the stomach in the GC-body and non-GC-body samples

**Figure 5:**
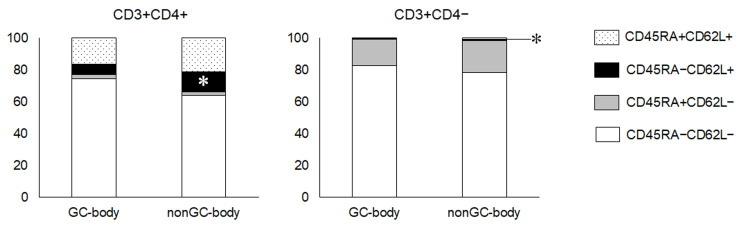
Subpopulation analysis based on CD45RA and CD62L expression. CD45RA^+^CD62L^−^ cells represent effector memory T cells, CD45RA^−^CD62L^+^ cells represent central memory T cells, and CD45RA^+^CD62L^+^ cells represent naïve T cells. *p < 0.05; t-test.

As described above, we discovered that patients with GC after *H. pylori* eradication had a relatively lower ratio of CD62L^+^/CD3^+^CD4^+^ in the lesser curvature of the stomach compared to those without GC. To explore the changes in lymphocyte composition in further detail, we separated the CD3^+^CD4^+^ and CD3^+^CD4^−^ cells into four subpopulations based on their CD45RA and CD62L level expression: CD45RA^+^CD62L^−^ cells were considered as effector memory T cells, CD45RA^−^CD62L^+^ cells as central memory T cells, CD45RA^+^CD62L^+^ cells as naïve T cells, and CD45RA^−^CD62L^−^ cells. As shown in Figure 5, the CD45RA^−^CD62L^+^/CD3^+^CD4^+^ fraction was lower in GC-body samples than in non-GC-body samples (6.3 ± 5.9% vs. 12.7 ± 10.0%). Although the ratio of CD45RA^+^CD62L^+^/CD3^+^CD4^−^ was also decreased in the lesser curvature of the gastric body of patients with GC after *H. pylori* eradication than in patients without cancer, the difference in the values was quite small (0.1 ± 0.3% vs. 0.9 ± 1.3%).

## Discussion

We conducted the present study to explore our hypothesis that lymphocyte composition in the stomach is different between patients with and without GC. To investigate the background factors associated with carcinogenesis in the gastric mucosa after *H. pylori* eradication, we enrolled patients with early GC rather than those with advanced-stage cancers. To our knowledge, this study is the first to investigate lymphocyte composition in the gastric mucosa after *H. pylori* eradication in patients with GC.

**Figure 6:**
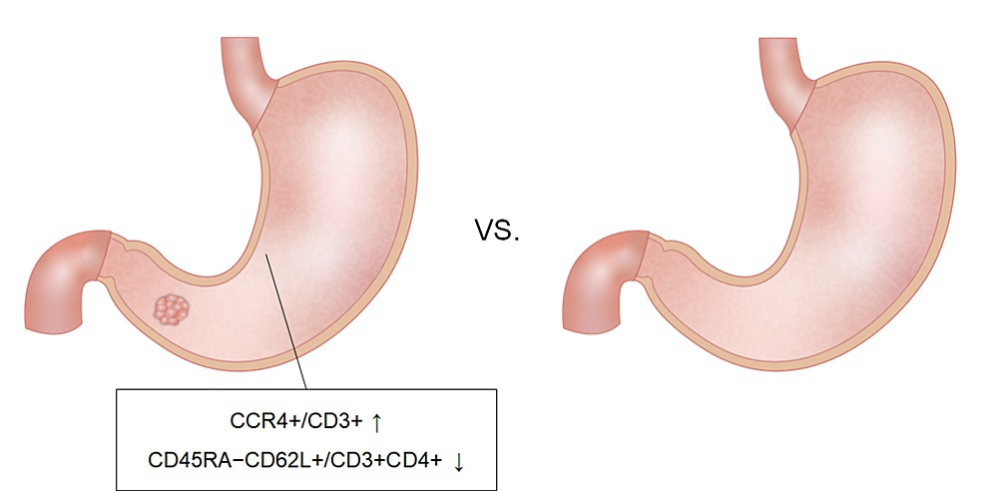
Images depicting differences in lymphocyte composition between the GC-body and non-GC-body samples. The proportion of CCR4^+^/CD3^+^ cells was larger, while the fraction of CD45RA^−^CD62L^+^/CD3^+^CD4^+^ cells was lower in the gastric mucosa of cancer patients than in that of non-cancer patients.

**Figure 7:**
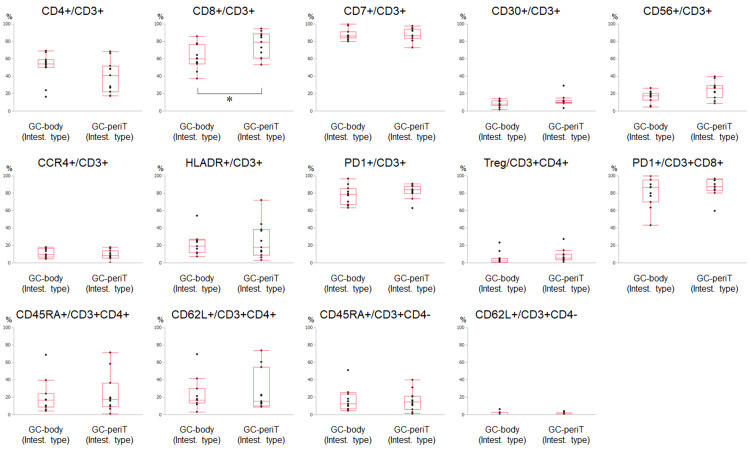
Flow cytometry results comparing the GC-peri-tumor (GC-periT) and GC-body samples of intestinal-type gastric cancer. The proportion of CD8+/CD3+ cells was higher in the GC peri-tumor sample. Regulatory T (Treg) cells were defined as CD3+CD4+CD25+CD127low/- cells. *p < 0.05 (Wilcoxon signed-rank test).

We found that the proportion of CCR4+/CD3+ cells was higher, while the fraction of CD45RA−CD62L+/CD3+CD4+ cells was lower in the lesser curvature of the gastric body of patients with early GC than in those without cancer (Figure 6). CCR4, also known as C-C chemokine receptor type 4 or CD194, is a receptor for CC chemokine ligands (CCLs) such as CCL2 (monocyte chemoattractant protein-1), CCL4 (macrophage inflammatory protein-1), CCL5 (regulated on activation, normal T-cell expressed and secreted), CCL17 (thymus and activation-regulated chemokine), and CCL22 (macrophage-derived chemokine). Increased levels of the chemokine receptor CCR4 have been reported in T lymphocytes in the *H. pylori*-infected stomach, suggesting a significant role of CCR4 in the accumulation of *H. pylori*-specific T lymphocytes in the infected stomach [16]. Our previous study revealed that continuous, prominent inflammation in the background gastric mucosa is a possible risk factor for the initiation of GC [9]. Therefore, prolonged inflammation in GC patients may mediate the recruitment of CCR4+ cells.

CCR4 is expressed in Tregs and Th17 cells [17,18]. Tregs are known to suppress antitumor immunity, thereby promoting carcinogenesis in certain settings [19]. Thus, CCR4+ Tregs may be involved in the development of GC after the eradication of *H. pylori*. However, in this study, no differences were observed in CD25+CD127low/−/CD3+CD4+ cells between GC-body and non-GC-body samples (5.8 ± 6.7% vs. 3.7 ± 4.6%, p = 0.425), which we defined as the Treg fraction. Another possible explanation for the larger proportion of CCR4+/CD3+ cells is the presence of CCR4+ Th17 cells. Th17 cells play critical roles in host defense, primarily by promoting immunity against extracellular pathogens at mucosal sites [20]. Th17 cells have paradoxical functions; they indirectly enhance antitumor immunity by inducing effector cell recruitment, activating tumor-specific cytotoxic CD8+ T lymphocytes, and transforming to the Th1 phenotype. Simultaneously, Th17 cells adopt a pro-tumor function by promoting angiogenesis, enhancing the proliferation of tumor cells, and transforming to the Treg phenotype [21].

Memory T cells are antigen-specific T cells that persist in the long term even after the exposure to the antigens has been eliminated. Upon re-exposure to a specific antigen, memory T cells are quickly converted into large numbers of effector T cells, thereby providing a rapid response [22,23]. Central memory T cells mainly exist in the T-cell region of the secondary lymphoid tissues. In our earlier study, central memory T-cell subsets were found to be increased in the rectum of patients with ulcerative colitis, suggesting that an enrichment of the central memory T lymphocytes in the gut mucosa is involved in the pathogenesis of ulcerative colitis [24]. The present study showed the opposite results; central memory T cells were decreased in patients with early GC after *H. pylori* eradication.

Although the exact roles of CCR4+/CD3+ cells and CD45RA−CD62L+/CD3+CD4+ cells are uncertain, the difference was significant between the GC and non-GC samples, whereas no significant difference was observed between the GC-peri-tumor and GC-body samples. These results suggest that lymphocyte subsets, including CCR4+/CD3+ cells and CD45RA−CD62L+/CD3+CD4+, are altered throughout the stomach of patients with GC. The mechanisms of GC development in association with gastric inflammation after *H. pylori* eradication have not been sufficiently investigated. We believe that focusing on CCR4+ Tregs, Th17 cells, and central memory T cells will help pave the way for in-depth research on the carcinogenesis of GC cells.

**Figure 8:**
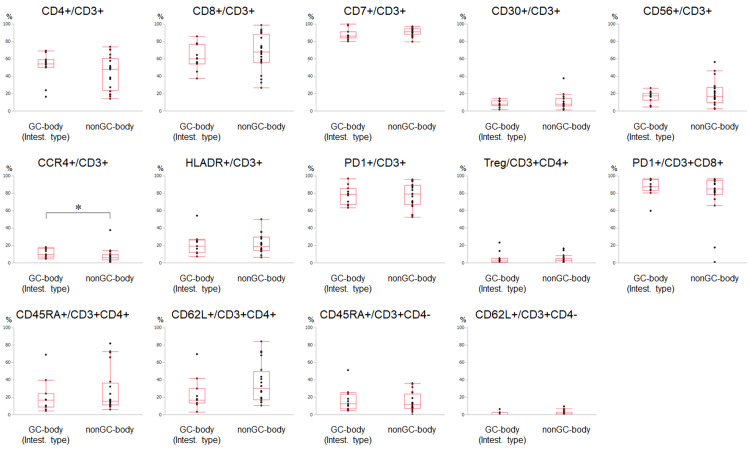
Flow cytometry results comparing GC-body samples of intestinal-type gastric cancer and non-GC-body samples. Regulatory T (Treg) cells were defined as CD3+CD4+CD25+CD127low/- cells. *p < 0.05 (Wilcoxon rank-sum test).

The analysis of the GC-peri-tumor and GC-body samples showed no statistically significant differences in the lymphocyte composition. However, subanalysis excluding diffuse-type cancers revealed that the percentage of CD8+/CD3+ cells was significantly higher in GC-peri-tumor samples than in GC-body samples (Figure 8 in Appendices). The intestinal type is the most common type of GC and is associated with *H. pylori* infection [25,26]. Thus, CD8+ cells may be involved in transforming normal stomach cells into cancerous cells under *H. pylori*-associated inflammation, followed by the intestinal metaplasia-dysplasia-adenocarcinoma sequence. Another possible explanation is that CD8+ cells increase in response to the emergence of GC cells. It has been known that CD8+ tumor-infiltrating lymphocytes play a key role in destroying tumor cells as immunological protection from neoplasia [27,28]. Thus, it is unclear whether a higher proportion of CD8+ cells in the peri-tumor mucosa within an area approximately 5 mm from the early GC lesion is the cause or the effect of the development of GC. We believe that a detailed analysis of CD8+ lymphocytes according to the Laurén classification will be valuable for addressing the role of these cells.

Our study had several limitations. First, the number of patients with early GC was relatively small because of the strict enrollment criteria. The number of patients was further reduced to maintain uniformity of patient characteristics between the groups; we excluded those patients who presented with factors that might have affected the gastric mucosa's lymphocyte composition, such as inflammatory bowel disease, autoimmune gastritis, immunosuppressive or anticancer drugs, and previously existing GCs. For instance, based on the difference in CD62L+/CD3+CD4+ cell ratio between the GC-body and non-GC-body samples (13.5%), and the pooled standard deviation of CD62L+/CD3+CD4+ cell ratio (20.8%), the required sample size to achieve a power of 80% and a level of significance of 5% (two-sided) was 38 in each group. Although the present study has low statistical power because of the insufficient patient number, these exploratory research results would help to define the number of groups in a further study. Second, we focused on T lymphocytes in the gastric mucosa, rather than on B lymphocytes. Our previous study revealed that inflammation of the background gastric mucosa is an independent risk factor for GC after *H. pylori* eradication. Immunostaining results showed that CD79 was strongly positive while CD3 was partially positive, suggesting that B lymphocytes were predominantly present than T lymphocytes [9]. Third, although flow cytometry analyzed T-lymphocyte markers using a panel of 13 commercially available antibodies, other markers may be involved in carcinogenesis. Therefore, further investigations with larger patient populations and/or animal studies on B-cell, functional studies including cytokine production (e.g., tumor necrosis factor α, interferon γ, and interleukins), T helper subsets (e.g., Th1, Th2, Th9, Th17, Th22, Tfh, Tr1, and Tregs), and T-cell clonality may reveal the true nature of lymphocytes in carcinogenesis in the gastric mucosa after *H. pylori* eradication.

## Conclusions

Our comprehensive analysis of lymphocyte composition in the gastric mucosa revealed that the proportion of CCR4+/CD3+ cells was larger, and CD45RA−CD62L+ cells among CD3+CD4+ cells were lower in the gastric mucosa of GC patients than in non-GC patients. Although the exact mechanism of the altered proportions of CCR4+/CD3+ and central memory CD4+ cells in the gastric mucosa of patients with cancer is unknown, focusing on lymphocytes in the gastric mucosa might help improve our understanding of carcinogenesis after *H. pylori* eradication. Moreover, differences in lymphocyte composition between cancer and non-cancer patients may be the key to distinguishing them on esophagogastroduodenoscopy screening.
